# Magnitude of prelacteal feeding practice and its association with place of birth in Ethiopia: a systematic review and meta-analysis, 2017

**DOI:** 10.1186/s13690-018-0308-y

**Published:** 2018-10-22

**Authors:** Wubet Worku Takele, Amare Tariku, Fasil Wagnew, Daniale Tekelia Ekubagewargies, Wondale Getinet, Lema Derseh, Degefaye Zelalem Anlay

**Affiliations:** 1 Department of Community Health Nursing, School of Nursing, College of Medicine and Health Sciences, University of Gondar, Gondar, Ethiopia; 2 Department of Human Nutrition, Institute of Public Health, College of Medicine and Health Sciences, University of Gondar, Gondar, Ethiopia; 3 Department of Nursing, College of Medicine and Health Sciences, Debre Markos University, Debre Markos, Ethiopia; 4 Department of Pediatrics and Child Health Nursing, School of Nursing, College of Medicine and Health Sciences, University of Gondar, Gondar, Ethiopia; 5 Department of Psychiatry, School of Medicine, College of Medicine and Health Sciences, University of Gondar, Gondar, Ethiopia; 6 Department of Epidemiology and Biostatistics, Institute of Public Health, College of Medicine and Health Sciences, University of Gondar, Gondar, Ethiopia

**Keywords:** Ethiopia, Place of birth, Prelacteal feeding, Systematic review and meta-analysis

## Abstract

**Background:**

Prelacteal feeding is one of the commonest inappropriate child feeding practice which exposes to malnutrition, infection, and neonatal mortality. However, there is no systematic review and meta-analysis that estimates the pooled prevalence of prelacteal feeding and its association with place of birth in Ethiopia. Therefore, this study aimed at investigating the magnitude of prelacteal feeding practice and its association with home delivery in the country.

**Methods:**

Primary studies were accessed through, HINARI and PubMed databases. Additionally, electronics search engines such as Google Scholar, and Google were used. The Joana Briggs Institute quality appraisal checklist was used to appraise the quality of studies. Data were extracted using Microsoft Excel spreadsheet. Heterogeneity between the studies was examined using the I^2^ heterogeneity test. The DerSimonian and Liard random-effect model was used. The random effects were pooled after conducting subgroup and sensitivity analyses. Publication bias was also checked.

**Results:**

A total of 780 primary studies were accessed. However, about 24 studies were included in the qualitative description and quantitative analysis of the prevalence of prelacteal feeding. To examine the association between home delivery and prelacteal feeding practice, only six studies were included. The prevalence of prelacteal feeding ranged from 6.1–75.8%. The pooled prevalence of prelacteal feeding among Ethiopian children was 26.95% (95% CI: 17.76%, 36.14%). The highest prevalence was observed in the Afar region. The pooled odds of prelacteal feeding among women who gave birth at home was increased by 5.16 (95% CI: 3.7, 7.2) folds as compared to those who gave birth at Health institutions.

**Conclusion:**

Prelacteal feeding practice in Ethiopia was found to be high. Home delivery was strongly associated with prelacteal feeding practice. Therefore, promoting institutional delivery and strengthening of the existing child nutrition strategies are recommended.

## Background

Exclusive breastfeeding (EBF) is one of the core indicators of Infant and Young Child Feeding(IYCF) practices providing many health benefits for the mother, as well as for her growing baby [1]. It reduces unnecessary expenses following health care service costs and infant feeding [2]. The World Health Organization (WHO) recommends exclusive breastfeeding for the first 6 months of age [3]. However, malpractices like prelacteal feeding is a bottleneck in ensuring optimal breastfeeding [4]. Prelacteal feeding is defined as the administration of any solid, semisolid, or liquid food other than breast milk to an infant during the first 3 days of birth [5]. The commonest foods given by Ethiopian women are butter, plain water, cow milk, sugar with water, and formula milk [6–8].

Prelacteal feeding might result in receiving insufficient breast milk, lactation failure, diarrhea, shortening of the breastfeeding duration, insufficient weight gain, and increased susceptibility to infection [9]. In addition, it is associated with late initiation of breastfeeding [10–12], which in turn leads to early neonatal morbidity and mortality [12–14]. Chronic and acute forms of under-nutrition are also the lethal effects of prelacteal feeding [15]. Gradually, early nutritional deficits are linked to impairments in intellectual performance, poor work capacity, and adverse reproductive outcomes [16]. Global risk assessment of suboptimal breastfeeding indicates that about 96% of all infant deaths in developing countries are attributable to inappropriate feeding occurring during the first 6 months of life [17]. Inappropriate breastfeeding including prelacteal feeding is responsible for around 45% of neonatal infection, 30% of diarrheal, and 18% of acute respiratory deaths in children aged below 5 years [18]. Furthermore, a recent study has proved that prelacteal feeding increases the risks of developing chronic noncommunicable diseases, diabetes mellitus, obesity, autoimmune disorders, and cardiovascular diseases in later ages [9].

Studies in different parts of the world reported that prelacteal feeding is a prevailing problem. For instance, it is reported to be about 73.3% in Vietnam [5], nearly a third (32·2%) in sub-Saharan Africa [19], 60% in Egypt [20], 31.3% in Uganda [21]. In Ethiopia, it ranged from 6.7–58.8% in different parts of the country [7, 22–25].

Cesarean section delivery and milk insufficiency were among perceived myths for prelacteal feeding [26]. An increased likelihood of prelacteal feeding was observed among mothers who had a lower educational level [27], attended fewer antenatal care visits, multiple births, delivering male infant as well as delivering the small sized baby [6]. Moreover, studies conducted in Ethiopia indicated that home delivery has a positive association with prelacteal feeding [28–30].

In cognizance of the severity as well as the wide spreading practices of inappropriate breastfeeding, Ethiopia has been devising different strategies including generating of health extension program, and working in collaboration with Non-Governmental Organizations (NGOs) [31, 32] in the areas of IYCF.

Despite various studies conducted in different parts of the country, the pooled national prevalence of prelacteal feeding is unknown. Likewise, results of the effect of home delivery on prelacteal feeding practice have been reported inconclusively. Therefore, the aim of this systematic review and meta-analysis was to determine the pooled national prevalence of prelacteal feeding and its association with home delivery. The findings of this study could help in improving the current IYCF (exclusive breastfeeding) practices in the country.

## Methods

### Study setting

This systematic review and meta-analysis were conducted in Ethiopian setting. Ethiopia is one of Africa’s populous nation having nearly 100,000,000 people with an area of 1,100,000 km^2^ making it the 27th largest country in the world. The country has diversified religion and cultures. In the country, traditional malpractices related to child feeding are critical problems affecting lots of children and women health. The country is working jointly with international partners to reduce maternal and child under-nutrition through the launching of IYCF, and National Nutrition Program (NNP). Likewise, reducing inappropriate child feeding and other maladaptive practices are amongst the health packages in which the country is working on. According to the 2016 Ethiopian Demographic and Health Survey (EDHS) report, the coverage of institutional delivery is reported to be 26% [33].

### Search strategies and quality appraisal

Articles were accessed through an electronics search of international databases, including, PubMed, Science direct, and HINARI. Similarly, Google Scholar and Google were used to retrieve extra articles including grey literature. Articles were searched and accessed by two reviewers (WWT, and DTE) using the following key terms, “prelacteal feeding practice”, “prevalence”, “associated factors”, “determinant factors”, “predictors”, “timely initiation of breastfeeding” “mothers”, and “Ethiopia”. These all key terms were searched by a combination of Boolean operators of “AND”, and “OR” as appropriate**.** Furthermore, additional articles were retrieved by cross referencing. For those studies having similar outcome of interest with the current objectives, their abstracts and the full-text were thoroughly reviewed. The quality of each article was appraised by three independent reviewers (WWT, AT, and FWS) using the Joana Brigg’s Institute (JBI) critical appraisal checklist for simple prevalence [34] and analytical cross-sectional studies [35] having nine and eight checklist items, respectively. Interpersonal scoring discrepancies during critical appraisal were resolved after a thorough discussion by reviewing the articles together. In case of persistent arguments during the quality assessment process, the mean scores of the three reviewers’ were calculated. All articles scored greater than 50%. This study was prepared by strictly following the standardized scientific writing format of the Preferred Reporting Items for Systematic Reviews and Meta-Analyses (PRISMA) guidelines having twenty-seven checklist items [36].

### Eligibility criteria

#### Inclusion criteria

Studies which described the prevalence of prelacteal feeding and risk factor (home delivery) were included.

There was no design, publication status, or language restrictions in this review as long as it was conducted in Ethiopia since 2008.

#### Exclusion criteria

Studies with poor definition of the outcome of interest, the difficulty of extracting necessary data, and inability to get the necessary details from the author(s) were excluded.

#### Outcomes of the study

The study contained two objectives namely, the magnitude of prelacteal feeding practice and the effect of birthplace on prelacteal feeding practice in Ethiopia. The prevalence was computed by dividing the number of women/caregivers reported as giving solid, semisolid, or liquid food within 3 days of birth by the total number of participants (sample size) and multiplied by 100. For the second objective, the odds ratio was computed by using the binary formula and it was estimated in the form of logs of the odds ratio.

#### Data extraction

The study was done from June, 03/2017 to September, 20/2017. Following the quality appraisal of all relevant articles, the necessary data were extracted by two reviewers (WWTand DZA) and recorded on pre-prepared standardized Microsoft excel spreadsheet data extraction format. For each eligible study, data were extracted based on the following characters; author’s first name, year of publication, sample size, number of study participants experienced the event, region, study setting, and the prevalence of prelacteal feeding practice. In addition to the above parameters, for the second objective, place of birth corresponding with experiencing status of the event (prelacteal feeding) was extracted.

### Data analysis

Once the necessary data were extracted and recorded on the Microsoft spreadsheet, for prevalence studies, the prevalence, logarithm of the prevalence, and standard error of the logarithm of the prevalence was computed. For the factor, the event (prelacteal feeding) versus exposure (place of delivery) status was extracted. Then, the odds ratio, logarithm of the odds ratio, and the standard error of the logarithm of the odds ratio were calculated. All extracted data were exported to STATA/SE version 14 (Stata Corp LLC, Texas, USA) for further analysis.

The presence of heterogeneity among studies was checked visually using forest plot, and objectively, using the I^2^ statistical heterogeneity test. For both objectives, in the fixed-effect model, it has been observed that the I^2^ was greater than > 50%. Thus, to determine the pooled estimates, the DerSimonian and Liard random-effect model was used [37]. To identify the influential study that caused variation, a sensitivity analysis was employed. Subgroup analysis was done by the region where primary studies were conducted, and by study setting. The existence of small studies publication bias was evaluated using the symmetry of funnel plots, and Egger’s regression test [38].

## Results

### Description of previous primary studies

**Fig. 1:**
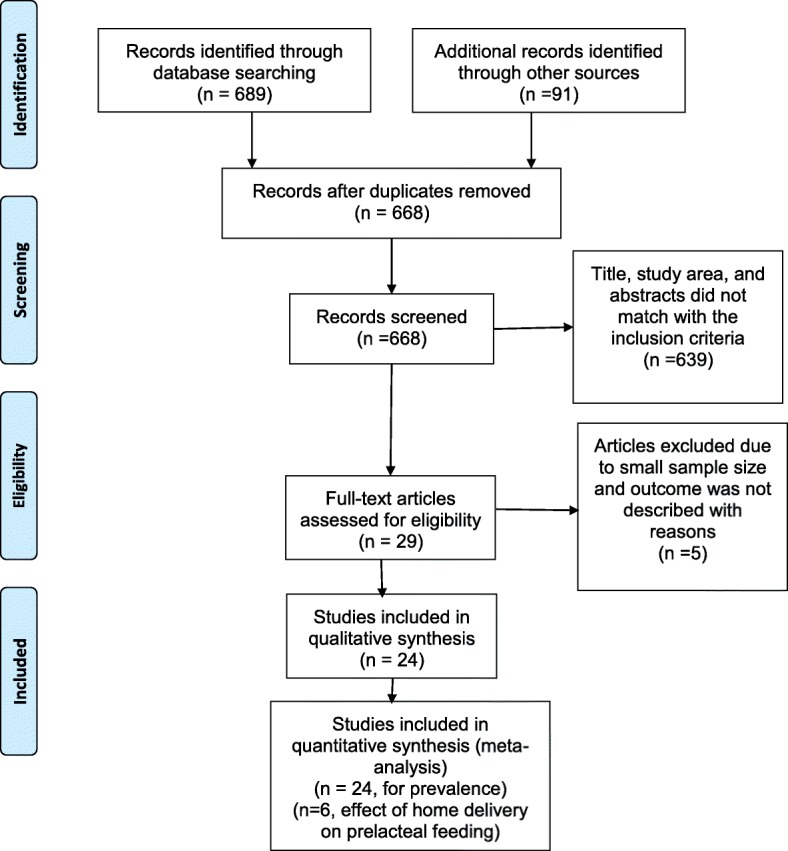
PRISMA flow diagram indicating the study selection procedure to include into the systematic review and meta-analysis, Ethiopia, 2017

Initially, a total of 780 articles were collected, of which 689 from PubMed and the rest from other electronics search engines mainly Google Scholar, Google, and HINARI (Fig. 1).

In this study, a total of 18,828 study participants were included. The sample size of included studies ranged from 6761 in Amhara Region, and 333 in Afar region [39]. Six studies were included to show the effect of home delivery on the prelacteal feeding (Table 1). All the included studies were written in English and cross-sectional by design.

**Table 1:** List of studies included to show the prevalence of prelacteal feeding practice among Ethiopian children, 2008–2017, a systematic review and meta-analysis, Ethiopia, 2017

S.no.	Authors name	Study setting	sample size	Prevalence of prelacteal feeding	Study quality
1	Tewabe T et al. 2016 [40]	Amhara	423	19.4	88.8%
2	Derso T et al. 2017 [41]	Amhara	6761	56.1	100%
3	Tilahun G et al. 2016 [42]	Amhara	416	15.9	100%
4	Gultie T et al. 2016 [26]	Amhara	548	24.3	100%
5	Liben L et al. 2016 [39]	Afar	333	16.8	100%
6	Egata G et al. 2013 [49]	Oromia	860	75.8	88.8%
7	Hailemariam TS et al. 2015 [25]	Oromia	594	6.7	100%
8	Lenja A et al. 2016 [48]	SNNP	396	6.1	100%
9	Mekuria G et al. 2015 [43]	Amhara	423	74.0	100%
10	Asemahagn et al. 2016 [44]	Amhara	346	20.2	100%
11	Bimirew D et al. 2016 [46]	Amhara	739	11.9	88.8%
12	Amare M et al. 2015 [7]	SNN	485	21.9	100%
13	Yenit M et al. 2017 [47]	Amhara	367	19.1	100%
14	Bililign N et al. 2016 [45]	Amhara	782	11.1	88.8%
15	Bekele Y et al. 2014 [30]	Harari	634	43.8	88.8%
16	Tariku A et al. 2016 [28]	Amhara	822	26.8	100%
17	Legese M et al. 2014 [29]	Amhara	630	38.7	100%
18	Teka B et al. 2015 [50]	Tigray	530	12.8	88.8%
19	Liben L et al. 2016 [56]	Afar	337	49.6	100%
20	Girma TS et al. 2008 [53]	Oromia	650	11.8	100%
21	Alemayehu Ay et al. 2015 [54]	Oromia	371	9.7	88.8%
22	Woldemichael B et al. 2016 [55]	Oromia	373	14.7	100%
23	Alemayehu M et al. 2014 [51]	Tigray	418	17.2	100%
24	Liben L et al. 2017 [57]	Afar	615	42.9	88.8%

With regarding regional distribution, about (45.8%) of the studies were conducted in Amhara region [26, 28, 29, 40–47]. The prevalence of prelacteal feeding practice ranged between 6.1% [48], and 75.8% [49] in South Nation Nationalities and Peoples (SNNPs) region and Oromia region, respectively. Concerning the distribution of studies, two of the studies were obtained from Tigray region [50, 51], a national report through EDHS [52], a study from Harari region [30], five from Oromia region [25, 49, 53–55], two from SNNPs region [7, 48], and three studies from Afar region [39, 56, 57]. However, there were no studies from Benishangule Gumize region, Somali region, and Addis Ababa, the capital of Ethiopia.

### The association of home delivery with prelacteal feeding practice

Regarding the association of place of birth and prelacteal feeding practice, six studies with a total of 3720 participants were included. Five studies, an institution based study from Harari region, Eastern Ethiopia [30], one national report [52], a community based study from Dabat Demographic and Health survey in Amhara Region [28], another a community based study from Amhara regiona [29], and a study from SNNPs of Ethiopia [7] reported that home delivery was positively associated with prelacteal feeding practice. On the other hand, a facility based study from northwest Ethiopia [47], and a community-based study from Amhara Region [45] showed that home delivery had no association with prelacteal feeding. The sample size of included studies ranged between 367, and 822 (Table 2). Except for a single study, all studies were published in reputable peer-reviewed journals.

**Table 2:** List of studies included to examine the effect of home delivery on prelacteal feeding practice among Ethiopian Children, 2008–2017, a systematic review and meta-analysis, Ethiopia, 2017

S.no.	Authors name	Region	Total sample size	Study setting	Study quality
1.	Amare M,et al. 2015 [7]	SNNPs^a^	485	Community	87.5%
2.	Yenit M et al. 2017 [47]	Amhara	367	Institutional	100%
3.	Bililign N et al. 2016 [45]	Amhara	782	Institutional	100%
4.	Bekele Y et al. 2014 [30]	Harari	634	Community	87.5%
5.	Tariku A et al. 2016 [28]	Amhara	822	Community	100%
6.	Legese et al. M2014 [29]	Amhara	630	Community	100%

^a^*SNNPs* South Nation Nationalities, and Peoples

### Pooled prevalence of prelacteal feeding practice

**Fig. 2:**
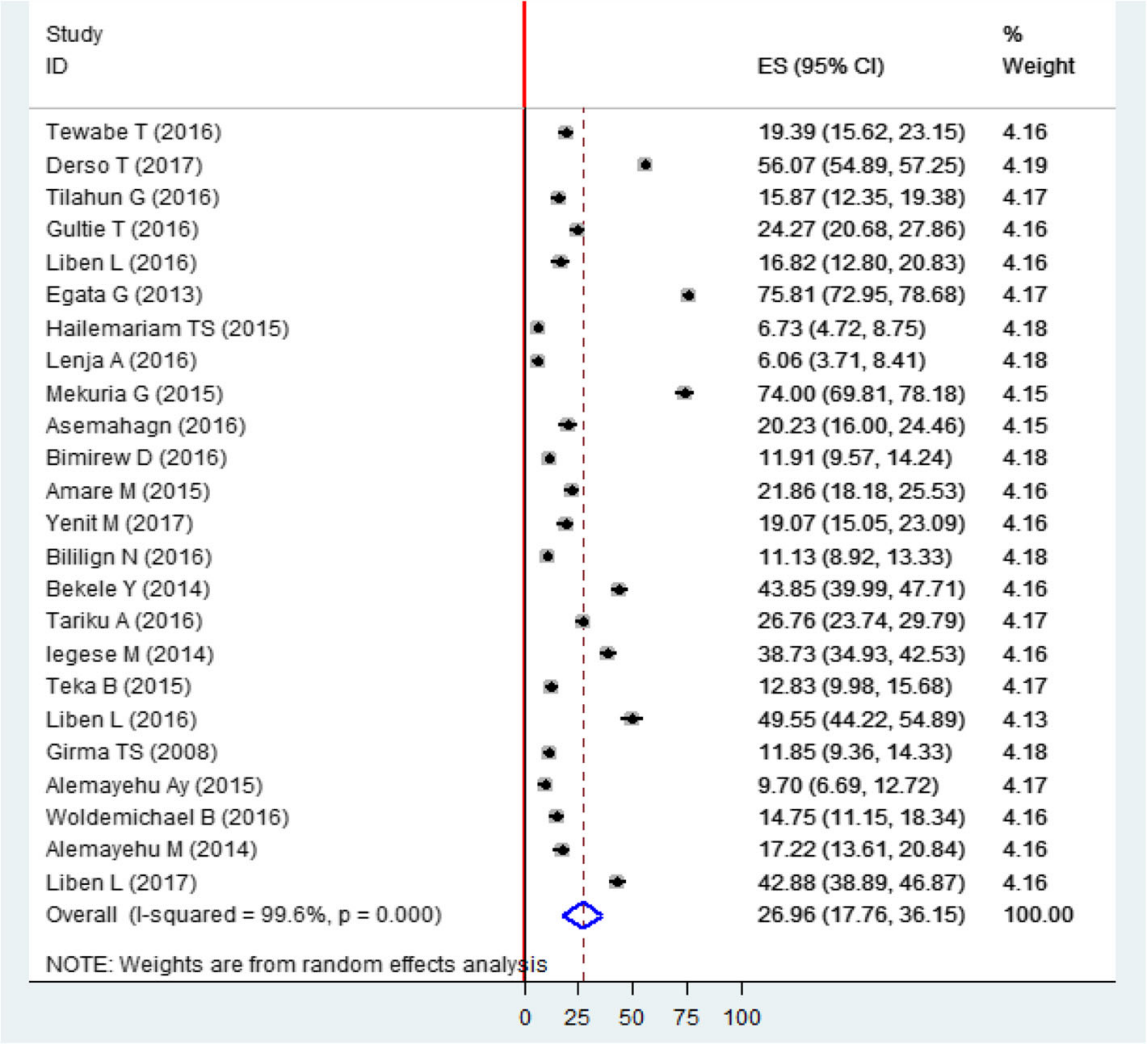
Forest plot of the pooled prevalence of prelacteal feeding among Ethiopian children using the random effect model, a systematic review and eta-analysis, Ethiopia, 2017

The pooled prevalence of prelacteal feeding practice in Ethiopia was 26.95% (95% CI: 17.76%, 36.14%). High heterogeneity was observed across the studies (I^2^ = 99.6%, *p* < 0.01) (Fig. 2). According to the sensitivity analysis, there was no single influential estimate that significantly accounted for it.

### Subgroup analyses

#### By geographical zones

**Fig. 3:**
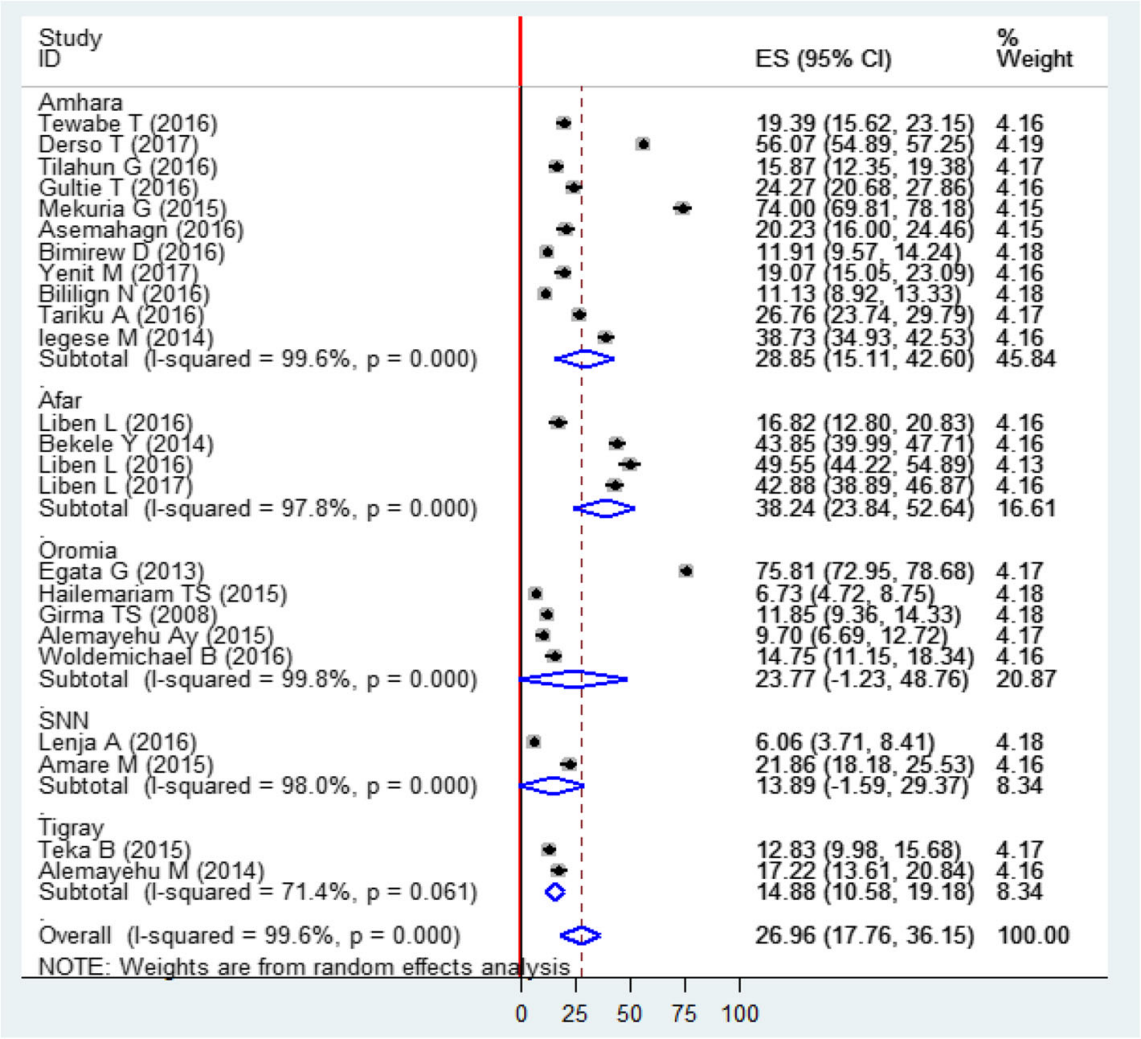
Forest plot of subgroup analyses by regions among Ethiopian children using the random effect model, a systematic review and meta-analysis, Ethiopia, 2017

The subgroup analysis by the region where studies were done showed that the highest prevalence is observed in Afar region, Eastern Ethiopia 38.2% (95% CI: 23.8%, 52.6%) and the lowest prevalence in SNNPs of Ethiopia 13.9% (95% CI: 1.5, 29.37) (Fig. 3).

#### By the study setting

Subgroup analysis by study setting showed that the overall prevalence of prelacteal feeding practice among community-based studies to be 26.5% (16.8CI, 36.3%) while for institution-based studies it was 31% (95% CI: I7.1%, 55.7%), and the observed difference was statistically significant (I^2^ = 99.6%, *p* < 0.01).

**Fig. 4:**
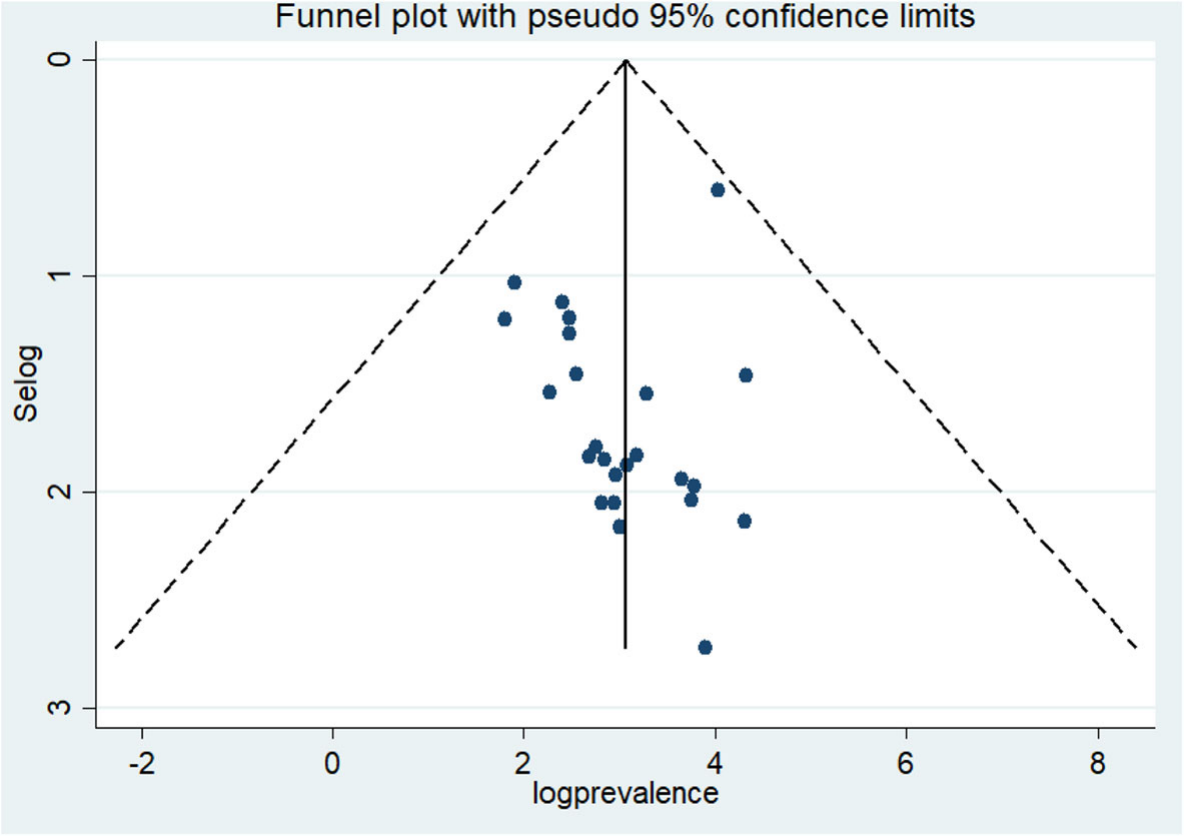
Funnel plot showing publication bias of prevalence studies among Ethiopian children, a systematic review and meta-analysis, Ethiopia, 2017

The funnel plot illustrated that there was symmetric distribution of studies (Fig. 4). Besides, the intercept (slop) of the Egger’s test graph was not significantly deviating from the origin (*p* = 0.47) revealing the absence of publication bias (Bias = 3.38; 95% CI: 2.42, 4.34).

##### Association of the place of birth with prelacteal feeding practice

**Fig. 5:**
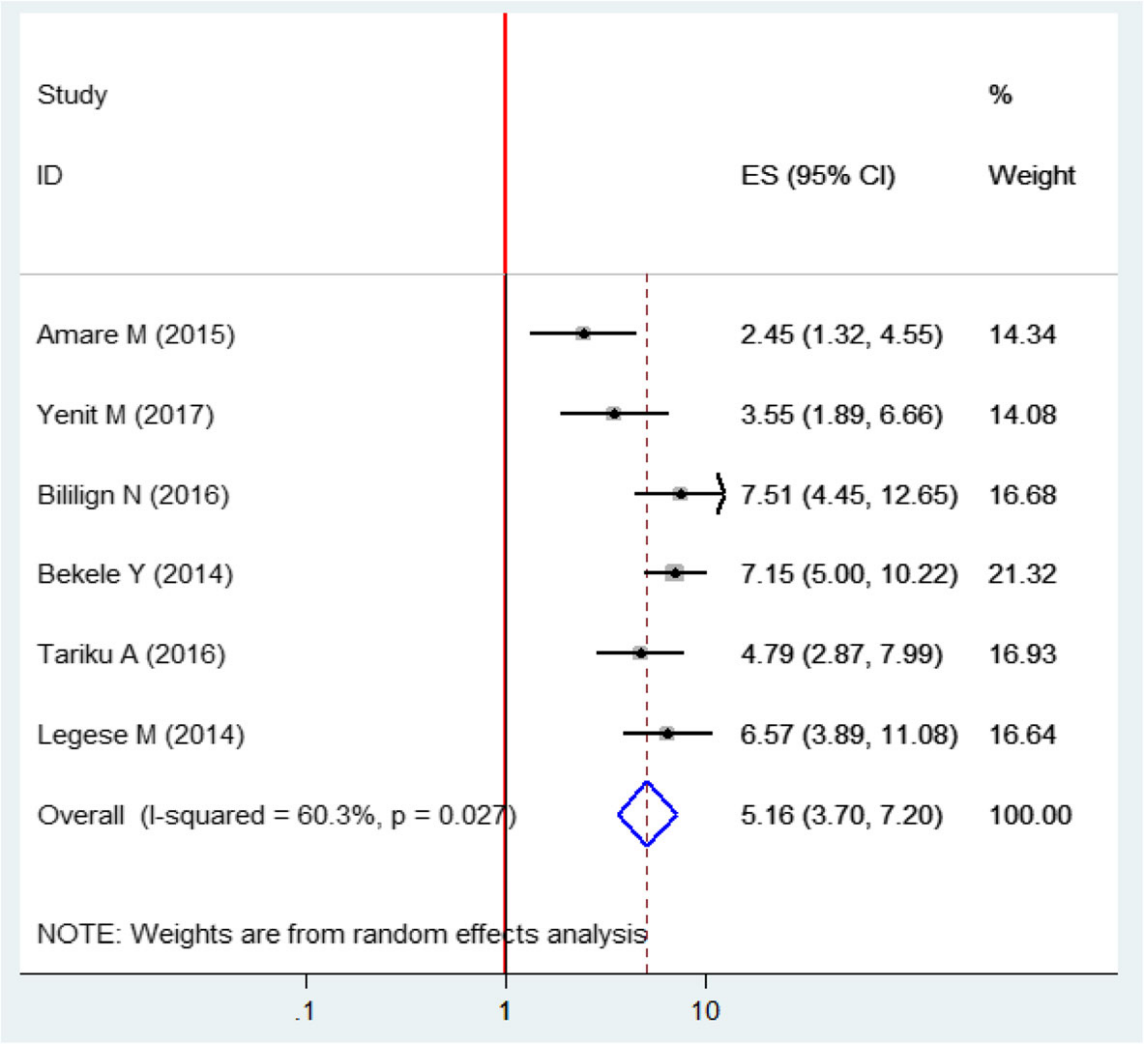
Forest plot of the pooled estimate of the effect of home delivery on prelacteal feeding among Ethiopian children using the random effect model, a systematic review and meta-analysis, Ethiopia, 2017

In the random effect model, weight was allocated based on the sample size and the effect size. The maximum (21.32%) and minimum (14.08%) weight were given for Bekele Y et al., and Yenit M et al., respectively. In this model, it has been observed that there was a positive statistically significant correlation between home delivery and prelacteal feeding practice. As an illustration, the pooled odds of experiencing prelacteal feeding among women who gave birth at home was increased by 5.16(95% CI: 3.7, 7.2) folds as compared to those who gave birth at health institutions. The I^2^ test suggested the presence of heterogeneity (I^2^ = 60.3%, *p* = 0.027) (Fig. 5).

**Fig. 6:**
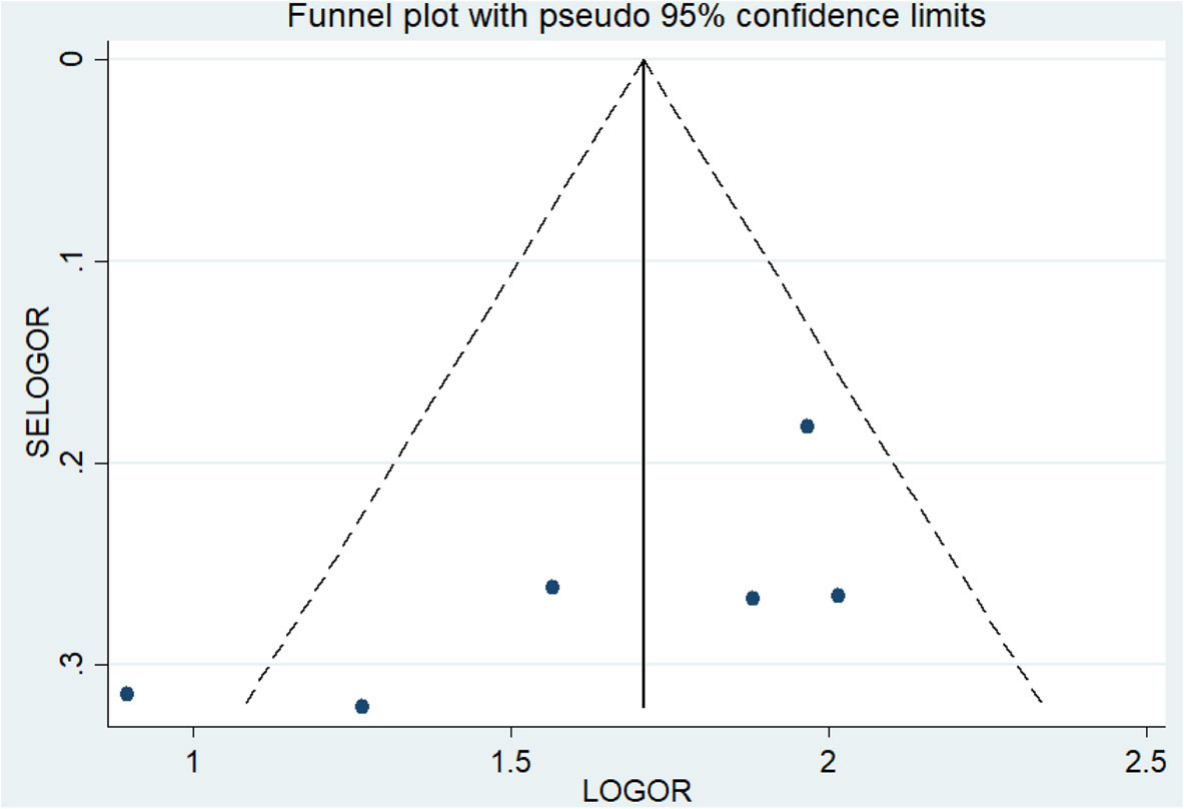
A funnel plot showing publication bias of studies in the effect of home delivery on prelacteal feeding among Ethiopian children, a systematic review and meta-analysis, Ethiopia, 2017

The sensitivity analysis of the six estimates also revealed that there was no any sign of influential effect size that affected the pooled estimate. According to the Egger’s regression test, publication bias was not a concern (*p*-value = 0.097) (Fig. 6).

## Discussion

The result of this systematic review exhibited that the highest (75.8%) prevalence of prelacteal feeding was observed in a study done in the year of 2011 in Oromia region [49], whereas the lowest (6.1%) was from SNNPs in 2015 [48]. The discrepancy could be due to the study participants’ educational status difference. The vast majority of study participants enrolled in Oromia’s study were illiterate as compared to SNNPs of Ethiopia study. Previous studies showed that being illiterate increased the likelihood of practicing prelacteal feeding [48]. The time difference when the two studies conducted might be considered as the source of the variation. The expansion of Community Based Nutrition (CBN) in the country level between this period may reduce the magnitude [58]. At the national level, the trend of prelacteal feeding has shown a decline from 27% in the year 2011 and 8% in 2016 [33].

The pooled prevalence of prelacteal feeding practice in Ethiopia to be 26.95% (95% CI: 17.76%, 36.14%). This implies that prelacteal feeding is a contributing malpractice factor which is interfering optimum breastfeeding in the country which needs strengthening of IYCF practice strategies. The likelihood of practicing prelacteal feeding among women who gave birth at home was five folds (AOR = 5.16, 95% CI: 3.7%, 7.2%) higher as compared to those women gave birth at Health facilities. This is suggesting that home delivery is not affecting only the health of women, rather it affects children’s feeding practice.

The current finding is congruent with studies conducted in Nepal (26.5%) [59], and a multilevel analysis study was done in twenty-two Sub-Saharan African countries including Ethiopia (32.2%) [6].

However, this finding is higher than the EDHS 2016 report (8%) [33] and twice higher than a survey done in Timor-Leste(12.3%) [60]. The EDHS report was publicized in 2016 while most of our primary studies were conducted in the years before 2016. Different IYCF interventions that were implemented by the Federal Minister of Health (FMOH) through the years could have helped in reducing prelacteal feeding over time [58]. The favorable traditional practice towards exclusive breastfeeding among Timorese mothers might contribute to reducing the magnitude [61]. The difference with Timor-Leste could be due to the fact that the study areas are located in a different segment of the world which could lead to differences in child feeding cultural practices.

The result of this study is far lower as compared to a nationwide study conducted in Vietnam in the year 2011 (73.3%) [5]. Misconception towards breastfeeding and other social norms among Vietnamese mothers could attribute to high prelacteal feeding [5]. Furthermore, breastfeeding practices may explain this discrepancy; for instance, In the year 2011, exclusive breastfeeding was reported to be 52% in Ethiopia [62], while 20% in Vietnam [63].

In the subgroup analysis, the burden of prelacteal feeding practice among regions was found to be different. The highest prevalence (38.24%) was noted in the Afar region. Good ANC service utilization and institutional delivery are factors which reduce prelacteal feeding. The lowest ANC and institutional delivery services in the region might describe the highest burden of prelacteal feeding [33]. During ANC visit mothers get advice about infant feeding and the importance of institutional delivery which further could improve infant feeding practices.

The finding of this study showed that home delivery practice was associated with prelacteal feeding. The pooled odds ratio of women who gave birth at their home was increased by five folds as compared to those who gave birth at health facilities. This finding is in agreement with a multilevel study in twenty-two Sub Saharan Africa including Ethiopia [6], and another multilevel study conducted in Ethiopia [24]. This is due to the fact that home delivery in Ethiopia is usually attended by traditional birth attendants who do not have the knowledge on the benefits of optimum breastfeeding and the harms following prelacteal feeding. On the other hand, Health facilities are places where early initiation of breastfeeding practice, the ill effects of early complementary feeding and ritual feeding practices are taught. At the same time, principles of IYCF practices, as well as the benefit of exclusive breastfeeding are sermonized by health professionals. This is supported by evidence from an Indian study that shows an increased prevalence of early initiation of breastfeeding and decreased the level of prelacteal feeding among mothers who gave birth at Health facilities than those who gave birth at home [64]. This review and meta-analysis study is the first of its kind in Ethiopia and searching key terms were broader which increased the number of included studies as a result, generalizability is possible with great confidence. Nevertheless, this study is not free of limitation. Accordingly, the study shared all the drawbacks of the random effect model. Because of lack of further subgroup definitions in the primary studies, the heterogeneity among studies couldn’t be resolved. Moreover, it would have been very good if the study considered other maternal and institutional factors which influence the practice of prelacteal feeding.

## Conclusion

Prelacteal feeding practice in Ethiopia was found to be high. The highest magnitude was observed in Afar region. Home delivery was found to be statistically correlated with prelacteal feeding practice. Therefore, advocating institutional delivery and creating awareness about optimal breastfeeding are strongly recommended. Moreover, special emphasis should be given in some regions where the problem is highly prevalent.

## Abbreviations

EDHS: Ethiopian Demographic Health Survey
IYCF: Infant and Young Child Feeding
JBI: Joana Brig’s Institute
NNP: National Nutrition Program
WHO: World Health Organization
